# A diagnostic algorithm for detection of urinary tract infections in hospitalized patients with bacteriuria: The “Triple F” approach supported by Procalcitonin and paired blood and urine cultures

**DOI:** 10.1371/journal.pone.0240981

**Published:** 2020-10-22

**Authors:** Kathrin Rothe, Christoph D. Spinner, Birgit Waschulzik, Christian Janke, Jochen Schneider, Heike Schneider, Krischan Braitsch, Christopher Smith, Roland M. Schmid, Dirk H. Busch, Juri Katchanov

**Affiliations:** 1 Institute for Medical Microbiology, Immunology and Hygiene, School of Medicine, Technical University of Munich, Munich, Germany; 2 Department of Internal Medicine II, School of Medicine, Technical University of Munich, Munich, Germany; 3 Institute of Medical Informatics, Statistics and Epidemiology, School of Medicine, Technical University of Munich, Munich, Germany; 4 Division of Infectious Diseases and Tropical Medicine, University Hospital, LMU Munich, Munich, Germany; 5 Department of Clinical Chemistry and Pathobiochemistry, School of Medicine, Technical University of Munich, Munich, Germany; 6 Department of Internal Medicine III, School of Medicine, Technical University of Munich, Munich, Germany; 7 School of Tropical Medicine and Global Health (TMGH), Nagasaki University, Nagasaki, Japan; 8 Clinical Research Department, London School of Hygiene and Tropical Medicine, London, United Kingdom; 9 German Centre for Infection Research (DZIF), partner site Munich, Munich, Germany; Northwestern University Feinberg School of Medicine, UNITED STATES

## Abstract

For acute medicine physicians, distinguishing between asymptomatic bacteriuria (ABU) and clinically relevant urinary tract infections (UTI) is challenging, resulting in overtreatment of ABU and under-recognition of urinary-source bacteraemia without genitourinary symptoms (USB). We conducted a retrospective analysis of ED encounters in a university hospital between October 2013 and September 2018 who met the following inclusion criteria: Suspected UTI with simultaneous collection of paired urinary cultures and blood cultures (PUB) and determination of Procalcitonin (PCT). We sought to develop a simple algorithm based on clinical signs and PCT for the management of suspected UTI. Individual patient presentations were retrospectively evaluated by a clinical “triple F” algorithm (F1 =“*f*ever”, F2 =“*f*ailure”, F3 =“*f*ocus”) supported by PCT and PUB. We identified 183 ED patients meeting the inclusion criteria. We introduced the term UTI with systemic involvement (SUTI) with three degrees of diagnostic certainty: bacteremic UTI (24.0%; 44/183), probable SUTI (14.2%; 26/183) and possible SUTI (27.9%; 51/183). In bacteremic UTI, half of patients (54.5%; 24/44) presented without genitourinary symptoms. Discordant bacteraemia was diagnosed in 16 patients (24.6% of all bacteremic patients). An alternative focus was identified in 67 patients, five patients presented with *S*. *aureus* bacteremia. 62 patients were diagnosed with possible UTI (n = 20) or ABU (n = 42). Using the proposed “triple F” algorithm, dichotomised PCT of < 0.25 pg/ml had a negative predictive value of 88.7% and 96.2% for bacteraemia und accordant bacteraemia respectively. The application of the algorithm to our cohort could have resulted in 33.3% reduction of BCs. Using the diagnostic categories “possible” or “probable” SUTI as a trigger for initiation of antimicrobial treatment would have reduced or streamlined antimicrobial use in 30.6% and 58.5% of cases, respectively. In conclusion, the “3F” algorithm supported by PCT and PUB is a promising diagnostic and antimicrobial stewardship tool.

## Introduction

Urinary tract infections (UTI) are among the most common types of infectious diseases in the United States and Europe [1, 2] and represent a frequent reason for Emergency Department (ED) visits, particularly in older adults [3, 4]. Currently, the growing awareness of asymptomatic bacteriuria (ABU) and asymptomatic leukocyturia and/or pyuria [5, 6] led to a crisis in definition and diagnosis of UTI [7]. Leukocyturia has a low positive predictive value for subsequent positive urine cultures and moreover is not clearly linked to infection even when positive cultures follow [8]. ABU is present in 10%-50% of elderly patients, depending on risk factors such as nursing home residency and catheterization [3, 4, 9]. Persistency of colonization despite antibiotic therapy has been shown [4]. These diagnostic challenges have serious clinical implications as health care providers tend to over-treat ABU [10] resulting in massive unnecessary antimicrobial use with side effects such as *Clostridium difficile* colitis, prolonged hospitalization and emergence of antimicrobial resistance [11].

Acute medicine providers are frequently faced with the task to establish the diagnosis of UTI, often with incomplete information at hand [1, 3, 12, 13]. On the one hand, patients without any genitourinary complaints might still have relevant UTI with bacteraemia (urinary-source bacteraemia, USB) [14]. On the other hand, linking worsening of mental status or undifferentiated fever directly to abnormal urinalysis will result in over-diagnosis of UTI followed by overuse of antimicrobials [7].

Considering treatment implications, there are three main clinical-laboratory scenarios in the ED setting:

First, asymptomatic leukocyturia and/or pyuria and ABU: no treatment recommended; Second, uncomplicated urocystitis (localized UTI): antimicrobial treatment -preferably with urine only activity- possible, but not compulsory;

Third, UTI with systemic involvement (SUTI): source control (e.g. urological management of obstruction) and treatment with systemic antimicrobial agents recommended;

It is challenging for acute care providers to reliably differentiate between SUTI, uncomplicated urocystitis and ABU. We hypothesized that the use of the inflammatory marker Procalcitonin (PCT) and obtaining blood cultures (BCs) might facilitate the diagnosis of relevant UTI in the ED setting.

The utility of obtaining BCs in the ED setting has been questioned due to frequent false positive results from contamination, low positivity rate and lack of impact on management in uncomplicated bacterial infections [15–17]. The role of PCT in management of bacterial infections in the ED is still a matter of debate [18]. PCT is a good predictor of pyelonephritis in children [19] and a small study showed that PCT at a cut-off <0.25ng/ml can help to rule out UTI with a high negative predictive value [20]. In one study, PCT levels correlated with bacteremia in patients with febrile UTI [21]. Therefore, the main question of the present study was whether PCT and BCs as a part of a clinical algorithm could help to differentiate between ABU, uncomplicated urocystitis and SUTI. Our hypothesis was, that obtaining paired urinary cultures and blood cultures (PUB) might support the diagnosis of SUTI in a subset of patients that would have been missed otherwise. We aimed to establish diagnostic criteria for selecting patients in whom SUTI should be suspected and in whom PCT and PUB should be obtained. We introduced a systematic approach to ED patients with suspected UTI based on the clinical presentation (3F “triple F” approach). We established an easy-to-use numerical score (3F “triple F” score). We hypothesized that application of the “3F” algorithm along with PCT and PUB could lead to a reduction of inappropriate antibiotic use in patients presenting with leukocyturia and/or bacteriuria in the ED.

## Materials and methods

We performed a retrospective analysis of patients presenting to a medical ED of a German university hospital between 1 October 2013 and 31 September 2018 in whom ED physicians suspected a systemic urinary tract infection and simultaneously collected at least one pair of BC, a urine culture and ordered PCT. Data extraction and analysis were performed using the HyBase® analysis system (epiNet AG, Bochum, Germany) as well as the clinic information system with laboratory testing results.

Inclusion criteria were age > 18 and suspected systemic urinary infection with simultaneous collection of urine culture with true positive bacteriuria, BC and PCT.

Mid-stream urine, single catheter urine or indwelling catheter urine collected at (re-)placement of catheter were cultured quantitatively and uro-pathogenic species regarded significant at 10^3^ colony-forming units (CFU)/ml in pure culture or mixed culture of no more than two pathogens. To discriminate true positive bacteriuria from contamination, microorganisms were classified as pathogenic species (such as *Escherichia coli*, *Staphylococcus saprophyticus*, *Klebsiella species (spp*.*)*, *Enterobacter spp*., *Proteus spp*., *Morganella morganii*, *Staphylococcus aureus* and *Enterococcus spp*.) in contrast to normal urogenital flora [22]. Initially, 403 Patients met the inclusion criteria. After exclusion of sterile urine cultures (n = 138) and contamination (n = 82), 183 cases were considered for further analysis.

Considering the difficulty in determining the clinical significance of coagulase negative *Staphylococci* [23] and viridans group *Streptococci* [24–26] in BCs, these isolates were reviewed separately on the basis of number of positive culture sets, the presence of intravascular devices, focus of infection and patients’ risk factors to discriminate true positive bacteremia from contamination.

The three “F-criteria” (Fever, Failure, and Focus) were derived from the current concepts of clinical presentation of an infection and the host’s response. We identified established criteria for systemic infection based on the existing literature [27–29] to use in the initial part of our algorithm to trigger further clinical / diagnostic investigations such as obtaining PCT and PUB. The first F represents “Fever”. Fever and chills have been shown to be a major criterion for predicting bacteremia in the ED [27]. Fever was the first sign defining sepsis on the basis of the systemic inflammatory response syndrome [28]. The second F “Failure” acknowledges the current concept of severe infection as a host’s response associated with organ failure [29]. The third F “Focus” is derived from the current definition of urinary tract infections [30] (**Table 1**).

**Table 1:** Definition of “3F” criteria for diagnosis of Urinary Tract Infection (UTI).

F1	“fever”	fever at triage, during stay in the ED, unequivocal statement of measured fever, rigors, or chills prior to presentation to the ED
F2	“failure”	failure/ dysfunction of any organ system:
		*brain* (e.g. altered mental state, behavioural changes, apathy)
		*kidney* (e.g. acute kidney injury, acute deterioration of kidney function)
		*circulation* (e.g. syncope, hypotension, tachycardia)
		*heart* (e.g. decompensated heart failure)
		*liver* (e.g. decompensated cirrhosis, acute-on-chronic liver failure)
		*gastrointestinal tract* (e.g. vomiting, diarrhoea)
		*metabolism* (e.g. metabolic acidosis with tachypnoe)
		*failure of functioning in the elderly* (e.g. acute malaise, fluctuating functioning, falls, delirium)
F3	“focus”	presence of specific focal symptoms of urinary tract infection as acute dysuria, urgency, frequency, suprapubic tenderness or costovertebral angle pain or tenderness

Abbreviations: ED emergency department.

A multiple logistic regression analysis exploring the effect of the chosen three factors on the presence of definite or probable SUTI in our cohort was performed and the Odds Ratios (ORs) show a detectable association for all three variables which seems to be most profound for “F3” and “F1” while variable “F3” is statistically significant associated with the presence of definite or probable SUTI (**S1 Table**).

The presence of a criterion was scored as “1” (e.g. F1 = 1), the absence as “0”.

Patients with pathological urinalysis results were divided into 3 groups: ABU, urocystitis and UTI with systemic involvement (**Table 2**).

**Table 2:** Definitions of clinical conditions associated with pathological urinalysis (leukocyturia, bacteriuria).

UTI with systemic involvement (SUTI)	Systemic involvement is defined as		
	a. bacteremia from urinary source with or without clinical symptoms of UTI		
	AND/ OR		
	b. features of systemic reaction as fever, organ failure or laboratory findings (e.g. Procalcitonin)		
	Genitourinary symptoms are defined as		
	a. acute dysuria, urgency, frequency and suprapubic tenderness (lower genitourinary symptoms)		
	AND/ OR		
	b. flank pain (upper genitourinary symptom)		
	bacteremic UTI (definite SUTI)	bacteremia due to UTI [if genitourinary symptoms are absent, referred to as urinary-source bacteremia (USB)]	
	probable SUTI	presence of genitourinary symptoms	
		AND	
		features of systemic involvement	
		AND	
		absence of an alternative focus for systemic reaction	
	possible SUTI	presence of genitourinary symptoms	absence of genitourinary symptoms
		AND	AND
		features of systemic involvement	features of systemic involvement
		AND	AND
		presence of an alternative focus	absence of an alternative focus
Urocystitis	presence of lower genitourinary symptoms		
	AND		
	absence of signs, symptoms or laboratory findings of systemic involvement from urinary source		
Asymptomatic bacteriuria (ABU)	absence of genitourinary symptoms		
	AND		
	absence of signs, symptoms or laboratory findings of systemic involvement from urinary source		

Abbreviations: UTI urinary tract infection, SUTI UTI with systemic involvement, USB urinary-source bacteremia.

Accordance was defined as the presence of the same microorganism in urine culture and BCs. For each microorganism isolated in the urine, this was calculated as percentage of cases with accordant bacteraemia out of all cases of true bacteraemia.

### Statistical analysis

The distribution of quantitative data is described by median (range). Qualitative data is presented by absolute and relative frequencies. Measures for diagnostic performance are presented together with their 95% confidence interval. The maximum value of Youden's index was used for selecting the cut-off for the PCT-value. Chi-square tests were used to analyse the association between two binary variables, and multiple logistic regression analyses were performed for exploring the effect of the three “F”-factors on the presence of definite or probable SUTI. All tests were conducted two-sided on a significance level of 5%. Statistical analyses were performed using IBM SPSS Statistics Version 25.0 (IBM Corp, Armonk, NY). Concordance was calculated [31] using the statistical software R (version 3.5.2; R Foundation for Statistical Computing, Vienna, Austria).

### Ethics approval and consent to participate

The Ethics Committee of the Technical University of Munich approved the protocol for this retrospective study and waived the need to obtain consent for the collection, analysis, and publication of the retrospectively obtained and anonymised data (approval No. 139/20 S).

## Results

### “Triple F” approach and diagnosis of urinary tract infections

During the study period, 183 ED encounters fulfilled the inclusion criteria, of whom ninety-one were female (50%), 182 where Caucasians (99.45%) and the median age of all patients was 73 years (IQR 59.5–80.75). The ED encounters resulted in hospital admission in 173 cases (94.54%), resulting in a median length of hospital stay of 8 days (range 1–126 days) with an all-cause in-house mortality of 6.56% (n = 12). As gender might have a potential influence on UTIs, we analysed the gender distribution of the three “F”-criteria and of the definite diagnoses but found no association between gender and the named variables (**S2 Table**).

Fever and/ or chills/ rigors at triage or in the history of the presenting complaint (F1) were documented in 140 patients (76.5%). Failure defined as any organ dysfunction attributable to UTI (F2) was detected in 115 patients (62.8%). Focal genitourinary symptoms as dysuria, suprapubic pain, flank tenderness (F3) were reported and documented in 54 patients (29.5%).

**Fig 1:**
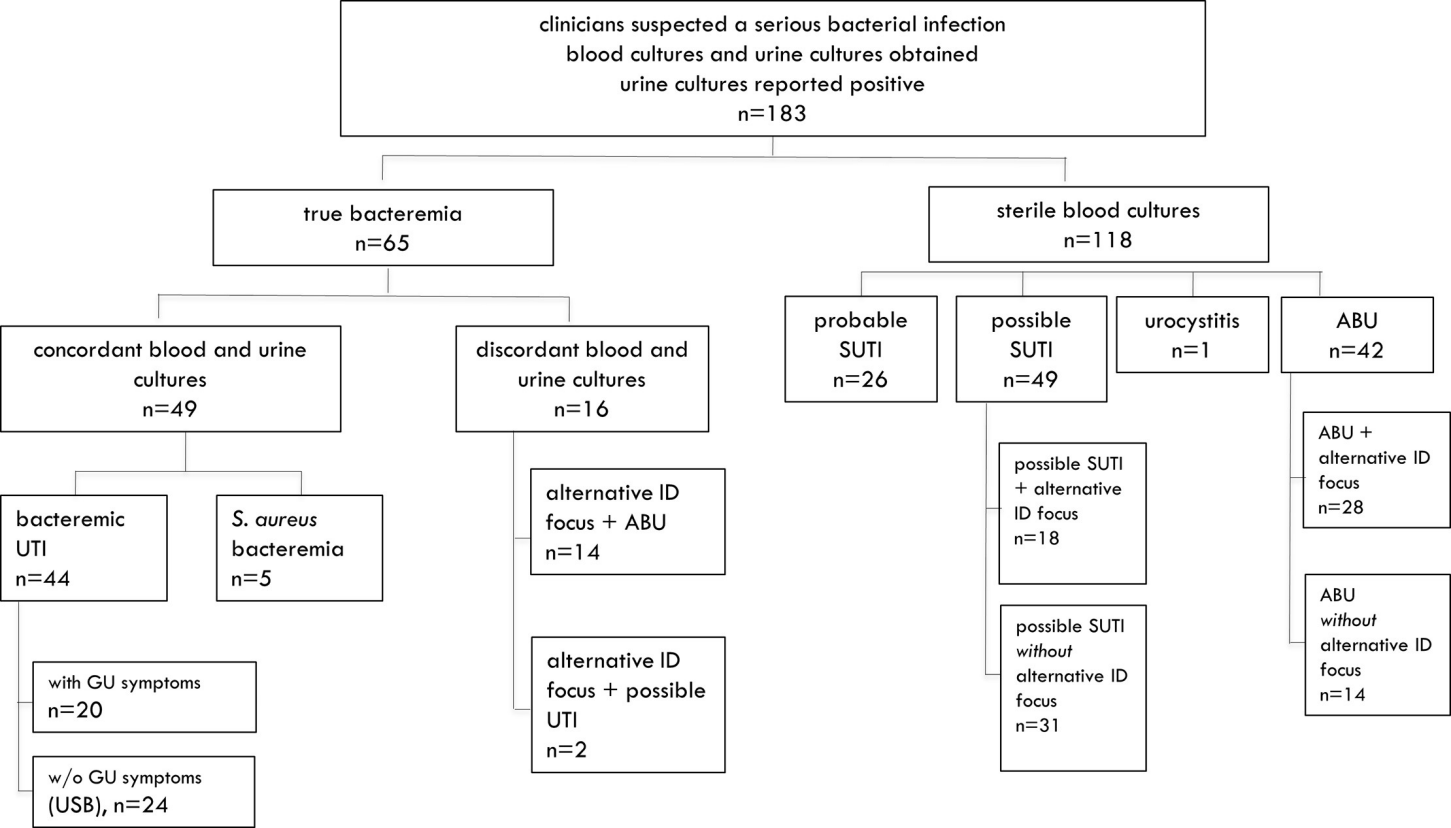
Study flow chart. Abbreviations: UTI urinary tract infection, USB urinary source bacteraemia, GU genitourinary, ID infectious diseases, ABU asymptomatic bacteriuria, SUTI UTI with systemic involvement.

The final diagnosis of bacteremic SUTI could be established in 44 patients (24.0%). The diagnosis of probable and possible SUTI was considered in 26 patients (14.2%) and 51 patients (27.9%), respectively. Urocystitis was diagnosed in one patient and ABU in 56 patients (30.6%) (**Fig 1**). An alternative infectious focus was identified in 67 patients: in five patients diagnosed with *Staphylococcus (S*.*) aureus* bacteriuria and in 62 patients diagnosed with possible SUTI (n = 20) and ABU (n = 42). The most common alternative infectious disease (ID)—diagnoses were pneumonia (n = 30), infection of totally implantable venous access device (port, n = 9), skin and soft tissue infection (SSTI, n = 9) and influenza (n = 4). The distribution of “F” criteria according to the “3F” approach is summarized in **Table 3**.

**Table 3:** Presence of “F” criteria and alternative ID diagnosis in patients presenting to the ED with bacteriuria.

Total cohort	F1 = 1	F2 = 1	F3 = 1	Established alternative ID focus
N = 183*	(fever)	(failure)	(focal symptoms)	
n (%)	n (%)	n (%)	n (%)	n (%)
Bacteremic SUTI (n = 44, 24.0%)	38 (86.4)	31 (70.5)	20 (45.5)	0 (0)
Probable SUTI (n = 26, 14.2%)	24 (92.3)	17 (65.4)	25 (96.2)	0 (0)
Possible SUTI (n = 52, 28.4%)	44 (84.6)	33 (63.5)	7 (13.5)	21 (40.4)
Urocystitis (n = 1, 0.51%)	0 (0)	0 (0)	1 (100)	0 (0)
ABU (n = 55, 30.6%)	32 (58.2)	29 (52.7)	0 (0)	41 (74.5)

Abbreviations: ID infectious disease, ED emergency department, ABU asymptomatic bacteriuria, SUTI UTI with systemic involvement.

*5 Patients with S. aureus bacteraemia and concomitant S. aureus bacteriuria were classified as established alternative ID focus but neither classified as UTI nor as ABU and are not listed as a separate row. In those five patients, fever (F1) was present in 2 patients, failure (F2) in all five patients and focal symptoms (F3) in one patient.

### Bacteriuria and accordant bacteraemia

In our cohort, in all patients with bacteriuria with relevant uropathogens, urine and blood cultures were drawn simultaneously (PUB). Mixed urine cultures with two pathogens were detected in 32 cases (17.5%). The microorganism most frequently isolated in the urine was *E*. *coli* (n = 94; 51.4%). Methicillin-sensitive *S*.*aureus* (MSSA) bacteriuria was detected in ten cases in which bacteraemia was detectable in 50%, highlighting the significance of *S*.*aureus* detection in urine as an indicator of a disseminated bacteremic infection as described previously [32, 33]. Methicillin-resistant *S*.*aureus* (MRSA) bacteriuria with accordant bacteraemia was detected in one only patient. Discordant bacteraemia was diagnosed in 16 patients (24.6% of all patients with bacteraemia). Accordance of bacteraemia in patients with bacteriuria was 85.3% for *Escherichia coli*, 60% for *Proteus mirabilis*, 50% for *Klebsiella pneumoniae* and *Pseudomonas aeruginosa* but only 16.7% for *Enterococcus faecalis* and 0% for *Enterococcus faecium* (**S3 Table**).

### Procalcitonin and detection of bacteraemia

PCT was obtained in all patients included in our study. The median value was 1 ng/ml (IQR 0–3 ng/ml). The value was *≥* 0.1 ng/ml in 149 patients (81.4%) and *≥* 0.25 ng/ml in 130 patients (71.0%). The diagnostic performance of a Procalcitonin cutoff of *≥* 0.25 ng/ml has been evaluated before [20, 34]. Also we confirmed this Procalcitonin cutoff in our cohort by calculating the Youden's index to select the optimum cut-off point for the PCT-value for probable SUTI (maximum value of Youden's index: PCT 0.25 ng/ml) and definite SUTI (maximum value of Youden's index: PCT 0.95 ng/ml). A PCT value *≥* 0.25 ng/ml was significantly associated with bacteraemia including accordant bacteraemia. Sensitivity and specificity of PCT for diagnosing true bacteraemia was 90.8% and 39.8% respectively. For accordant bacteraemia, sensitivity was 95.9% and specificity 38.1% (**Table 4**).

**Table 4:** Utility of PCT as predictor for accordant bacteraemia in all patients with bacteriuria (N = 183).

	Any true bacteraemia	Accordant bacteraemia
PCT < 0.25 ng/l (53 cases)	6 (11.3)	2 (3.8)
n(%)		
PCT ≥ 0.25 ng/l (130 cases)	59 (45.4)	47 (36.2)
n(%)		
NPV PCT < 0.25 ng/l	88.7% (77.0%, 95.7%)	96.2% (87.0%, 99.5%)
(95% confidence interval)		
PPV PCT ≥ 0.25 ng/l	45.4% (36.6%, 54.3%)	36.2% (28.0%, 45.0%)
(95% confidence interval)		
Sensitivity of proposed PCT dichotomisation	90.8% (81.0%, 96.5%)	95.9% (86.0%, 99.5%)
(95% confidence interval)		
Specificity of proposed PCT dichotomisation	39.8% (30.9%, 49.3%)	38.1% (29.8%, 46.8%)
(95% confidence interval)		
Concordance: Cohen´s Kappa	0.22 (0.14, 0.31)	0.25 (0.15, 0.35)
(95% confidence interval)		

Abbreviations: PCT Procalcitonin; ED emergency department; PPV positive predictive value; NPV negative predictive value.

### Bacteremic SUTI

Bacteremic SUTI was present in 44 patients. In these cases, focal signs of genitourinary infection such as dysuria were documented in 20 patients (45.5%) and signs of failure were detected in 31 patients (70.5%). We applied the “3F” approach combined with PCT to our cohort to investigate which combinations of “F”-parameters and PCT were associated with bacteremic SUTI (**S4 Table**). The highest rate (61.9%) of bacteremic SUTI was present in patients diagnosed with “fever” (F1), “failure” (F2) and “focal symptoms” (F3), i.e. “3F” score = 3 and PCT ≥ 0.25 pg/ml. In afebrile patients with no focal symptoms but with documented “failure” (F2 only) the rate was 27.8% if PCT was above 0.25 pg/ml.

### Algorithm for diagnosing UTI based on the “3F” system

**Fig 2:**
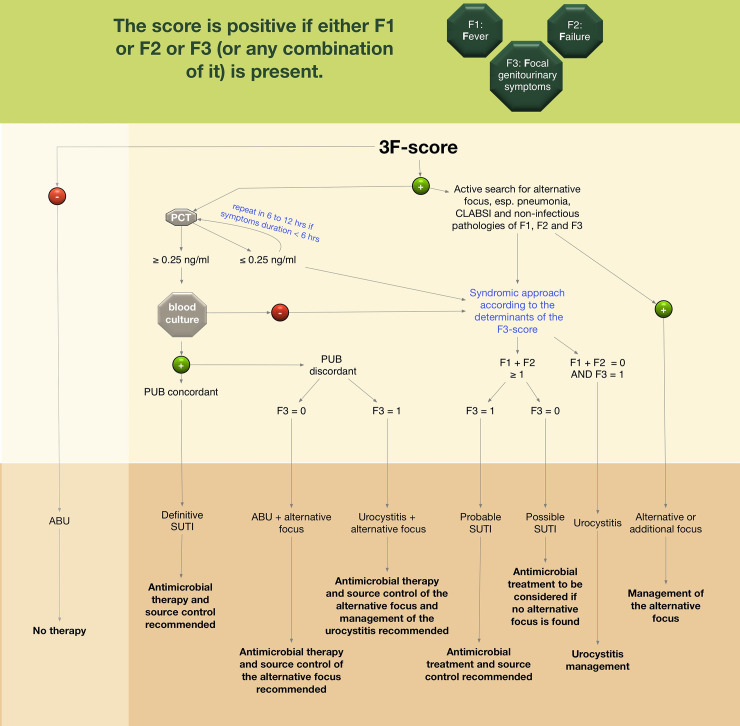
Proposed algorithm for identification and classification of urinary tract infections and asymptomatic bacteriuria in hospitalized ED patients with bacteriuria. The final diagnosis will be established after the completion of the algorithm. If BC obtained, the final diagnosis might be evident after 72 hours. *PCT at triage/ admission acceptable if duration of symptoms > 6 hours, otherwise repeat for re-evaluation if negative and highly suspected infection. Abbreviations: ABU asymptomatic bacteriuria, BC blood cultures, SUTI urinary tract infection with systemic involvement, PCT Procalcitonin, PUB paired urinary cultures and blood cultures, SSTI skin and soft tissue infection, CLABSI catheter-related bloodstream infection.

We propose an algorithm, using the “3F” scoring system and PCT to diagnose UTI in the ED (**Fig 2**). The algorithm is based on data of ED patients directly admitted to the hospital and therefore might only apply to this patient population.

The individual presence of F1 or F2 as isolated clinical findings does not significantly correlate with the presence of definite or probable SUTI but using these criteria in the initial part of our algorithm to trigger further clinical / diagnostic investigations such as obtaining PCT and PUB allows distinction between SUTI and ABU after completion of the algorithm.

### Antimicrobial use

Empiric antimicrobial therapy was initiated by the ED physician in 175 patients in the ED-setting (95.6%), as in 6 patients no antimicrobials were initiated and for 2 patients information on antimicrobial use was missing. In those 175 patients, the most used antimicrobial for empiric therapy was piperacillin/tazobactam (n = 94, 53.7% of all patients with antibiotic therapy), followed by ampicillin/sulbactam (n = 36, 20.6%) and meropenem (n = 21, 12.0%). An empiric combination regimen of 2 drugs was initiated in 15 patients (8.6% of all patients with antibiotic therapy). In one patient, 3 drugs were initiated (meropenem, linezolid and moxifloxacin). The most frequently chosen combination partner was linezolid (n = 8), followed by vancomycin (n = 4).

### Application of the “3F” algorithm to our cohort and reduction of unnecessary blood cultures and inappropriate antimicrobial use

Using the proposed algorithm, no BCs would have been obtained in patients with “3F” score = 0 (n = 14) and/ or PCT < 0.25 pg/ml (n = 53, in which n = 6 also had “3F” score = 0) resulting in a 33.3% (61/183) reduction of BCs.

Fourteen patients were diagnosed with ABU without an alternative ID focus. In 41 patients with ABU an alternative focus was detected, predominantly pneumonia and SSTI. Among unstable patients with suspected SUTI, an empiric therapy with piperacillin/tazobactam was commonly initiated, but for community acquired pneumonia and skin and soft tissue infections ampicillin/sulbactam was the antimicrobial of choice. Thus, the detection of an alternative ID focus could result in administration of targeted antimicrobial therapy with a narrower spectrum. If the threshold to start treatment empirically was set as diagnosis of possible UTI, then the use of our algorithm would result in withholding antimicrobials in 14 patients and using a potentially narrower spectrum antimicrobial in 41 patients, resulting in 55 patients (30.1%) eligible for an ABS intervention in our cohort. If the threshold to start treatment empirically was set as diagnosis of probable UTI, then the use of our algorithm would result in withholding antimicrobials in 45 patients and using a narrower spectrum antimicrobial in 62 patients (107 patients, 58.5%) (**S1 Fig, S5 Table**).

## Discussion

### Presentation of UTI in the ED and classification of bacteriuria

In patients presenting to the ED with leukocyturia/ bacteriuria it is crucial to distinguish between asymptomatic bacteriuria, uncomplicated urocystitis and UTI with systemic involvement, e.g. parenchymatous kidney involvement or bacteremia. The terms “febrile UTI” and “urosepsis” have been used to designate patients with systemic involvement. Our study shows that these categories are of limited clinical use even applied to bacteremic UTI as a definite UTI with systemic involvement.

Fever at triage and history of chills/ rigor were absent in almost 15% of patients with bacteremic UTI. Only 45% of all patients complained of focal signs of a urinary tract involvement. The term “urosepsis” should be avoided as the definition of sepsis has been undergoing adjustment and change [35]. We suggest the term “UTI with systemic involvement (SUTI)” for all patients with relevant systemic infection originating from the urinary tract.

Our study adds to the growing body of evidence that bacterial colonization of the urinary tract and resulting bacteriuria is a frequent phenomenon, especially in older patients. In our study, ABU were diagnosed in nearly a third of all patients with bacteriuria. Bacteremic UTI as a presentation of a definite SUTI was diagnosed in a quarter of patients with bacteriuria. In nearly half of the cohort, the diagnosis of SUTI was not unequivocal. Over-diagnosing this group would lead to overtreatment with antimicrobials and omission of alternative diagnoses. However, under-diagnosis might result in missing an important source of infection. To address this clinical dilemma, we propose the terms “probable SUTI” and “possible SUTI”. The “antimicrobial sparing effect” of our algorithm depends on the threshold to start UTI-treatment in relation to certainty of diagnosis. Depending on clinical severity, the attending clinician can decide which threshold to use, and initiate or withhold antibiotics accordingly.

### Obtaining blood cultures to diagnose UTI

Presentation of UTI with mild and unspecific clinical signs is not uncommon both in men and women [36]. Our study showed that in 55% of cases with definite UTI (i.e. accordant bacteremic UTI) genitourinary symptoms were absent or not identifiable. BCs were the only diagnostic tool to differentiate between ABU and SUTI in these patients. More than 50% of the patients with bacteremic UTI presented with undifferentiated fever and/ or failure. Our study highlights the importance of collection of PUB as a valuable diagnostic tool for evaluating patients with undifferentiated fever and/ or failure in the ED. Obtaining BCs in patients with suspected UTI can be helpful beyond its usefulness as a diagnostic tool as diagnosing bacteremia in UTI might help to stratify the risk of patients: Mortality rate for patients hospitalized for UTI varies from 7.5 to 30% [37, 38], bacteraemia is present in 15–40% of patients with complicated UTI [38] and bacteremia is an independent risk factor for mortality [39].

For patients with pyelonephritis, studies have shown robust conordance of urine cultures and BCs [40] and authors argue therefore that results of BCs do not change clinical management [41]. Contrary to these studies, our results point out the utility of PUB. BCs led to establishment of an alternative focus in 16 patients and detection of *S*. *aureus* bacteraemia in 5 patients. In patients with febrile UTI, indwelling urinary catheters, malignancy and antimicrobial treatment were risk-factors for bacteraemia with an uropathogen that could not be cultured in the urine simultaneously. Of interest, these patients with discordant culture results had a 4-fold higher mortality risk over a 90-day follow-up [42]. In 6 patients with accordant bacteremic bacteriuria in our cohort, two pathogens were detected in the urine but only one of these pathogens in the BC. Therefore, obtaining BCs can help to identity the dominant most relevant uro-pathogen.

The accordance for bacteraemia varied for different pathogens. The accordance for enterococci was very low. In 21.8% of enterococcal bacteriuria, a bacteraemia with a different microorganism from another source could be detected as the causative microorganism. The presence of enterococci in the urinary tract often represents ABU or colonization without therapeutic implications [10, 43]. UTI due to enterococci is associated with anatomical -or functional uropathy and with urinary tract catheterization or instrumentation [44]. Our results support the finding that enterococcal UTIs are associated with low complication rates and the use of antibiotics in enterococcal bacteriuria without risk factors could be restricted [10].

### Ordering Procalcitonin to diagnose UTI

The use of PCT in the work-up of UTI has become of increasing interest to emergency medicine providers [20, 45]. While PCT levels do correlate with bacteremia, C—reactive protein does not show any correlation with relevant clinical parameters in patients with febrile UTI [21].

Observational studies showed that PCT can predict bacteremia in patients with febrile UTI with dichotomized PCT of 0.25 μg/l as a robust surrogate marker [34].

In our “3F” algorithm, we use PCT as a trigger to obtain BCs for PUB as a diagnostic tool. PCT was particularly helpful in diagnosing bacteremic UTI in bacteriuric patients presenting with isolated failure and isolated fever. In these patients the rate of accordant bacteremic UTI was 27.8% and 22.7%, respectively if PCT was > 0.25 pg/ml. Obtaining PCT might also be a useful prognostic tool. Although a single value PCT does not correlate well with severity of sepsis, mortality or outcome in ED patients with pyelonephritis [46, 47], initial PCT levels are a predictor of progression to septic shock in patients with acute pyelonephritis [48].

### Using the algorithm as an ABS tool

Early evaluation of PCT and BC in the ED of patients with febrile UTI could be useful in the context of ABS. Of note, patients with uncomplicated UTI who had BC sampling in the ED had a shorter hospital stay [38]. Furthermore, for respiratory tract infections and UTIs the use of PCT has been shown to result in lower antibiotic consumption [49, 50].

ABU is a common phenomenon [3, 4] therefore general initiation of antimicrobial therapy in bacteriuria leads to unnecessary antibiotic prescriptions. Our algorithm assists the ED physician in classification of abnormal urinalysis as SUTI, urocystitis, or ABU. Introducing the terms “probable SUTI” and “possible SUTI” and defining the threshold to start antimicrobial treatment as part of an ABS-intervention could reduce the use of broad-spectrum antimicrobials in bacteriuric ED patients.

This study has several limitations. It is a single-center study based on the retrospective analysis of laboratory data. However, the heterogeneous population of a city-center-based ED with a large catchment area may allow generalization to other EDs. Due to the retrospective design of the study, no additional details on the quality of BCs were available and no clinical endpoints were available. The number of ED patients with UTI in our cohort represents only a fraction of all ED presentations due to the strict inclusion criteria. Our cohort represents severe presentations of UTI in the ED emphasizing the clinical significance of our findings and the value of the proposed algorithm for patients who will be eventually admitted for in-patient care.

## Conclusions

Our “3F” algorithm supported by PCT and paired urine cultures and BCs is a valuable tool to diagnose UTI with systemic involvement in hospitalized ED patients. It is particularly helpful to diagnose urinary-source bacteraemia in the absence of genitourinary symptoms. The simple algorithm supports the ED physician directly in terms of diagnostic evaluation and clinical management and also takes afebrile presentations and organ dysfunction as a sole manifestation of systemic infection into account. Moreover, the use of PCT with a predefined threshold could lead to a reduction of unnecessary BCs and therefore provide a timely, cost-effective and safe option for ED patients. The “3F” algorithm can also be applied as an ABS tool. Introducing the terms “probable SUTI” and “possible SUTI” and defining the threshold to start antimicrobial treatment might lead to reduction and streamlining of antimicrobial use in bacteriuric ED patients.

## Supporting information

S1 Fig Potential ABS interventions using different thresholds to initiate antibiotic treatment: While probable SUTI and bacteremic SUTI generally require systemic treatment, antimicrobials could be withheld in cases of possible SUI and ABU under close clinical monitoring until definite diagnosis is established. Abbreviations: ABU asymptomatic bacteriuria, SUTI urinary tract infection with systemic involvement, ABS antimicrobial stewardship, ID infectious diseases.
(TIF)

S1 Table Multiple logistic regression analysis exploring the effect of the chosen three “F”-criteria on the presence of definite SUTI or probable SUTI. (DOCX)

S2 Table Analysis of the association between gender and presence of “F”-criteria and definite diagnoses. (DOCX)

S3 Table Bacteraemia and accordance of paired blood and urine cultures in patients with bacteriuria. (DOCX)

S4 Table Diagnosis of bacteremic UTI (as surrogate of definite systemic UTI) in relation to “3F” score and PCT. F1: fever, F2: failure, F3: focal symptoms.
(DOCX)

S5 Table Application of the “3F” algorithm as an antimicrobial stewardship (ABS) tool. (DOCX)

## Abbreviations

ABS: Antibiotic stewardship
BC: Blood Culture
ED: Emergency Department
UTI: Urinary tract infections
ABU: Asymptomatic bacteriuria
USB: Urinary source bacteraemia
PCT: Procalcitonin
spp: species
SUTI: UTI with systemic involvement
PUB: Paired urinary cultures and blood cultures
CFU: Colony-forming units
ID: infectious disease
SSTI: skin and soft tissue infection
