# Supplementary figures and images for: A diagnostic algorithm for detection of urinary tract infections in hospitalized patients with bacteriuria: The “Triple F” approach supported by Procalcitonin and paired blood and urine cultures
Source: PLoS One. 2020 Oct 22;15(10):e0240981. doi: 10.1371/journal.pone.0240981 (PMC7580978; doi:10.1371/journal.pone.0240981)

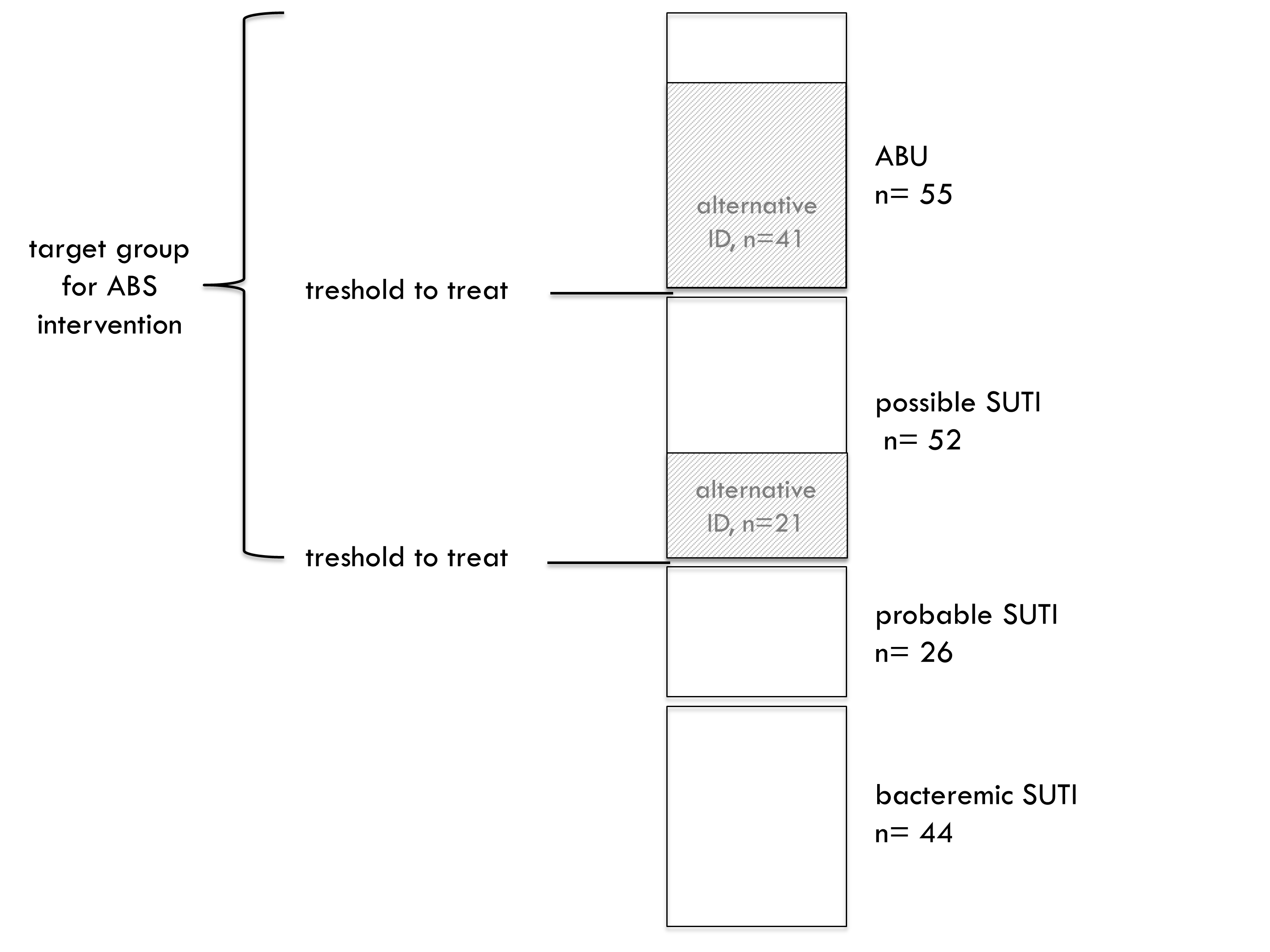

Supplement: S1 Fig — Abbreviations: ABU asymptomatic bacteriuria, SUTI urinary tract infection with systemic involvement, ABS antimicrobial stewardship, ID infectious diseases. (TIF) [file pone.0240981.s001.tif]
