# Supplementary material for: A diagnostic algorithm for detection of urinary tract infections in hospitalized patients with bacteriuria: The “Triple F” approach supported by Procalcitonin and paired blood and urine cultures
Source: PLoS One. 2020 Oct 22;15(10):e0240981. doi: 10.1371/journal.pone.0240981 (PMC7580978; doi:10.1371/journal.pone.0240981)
Supplement: S1 Table — (DOCX) [file pone.0240981.s002.docx]

**S1 Table**. Multiple logistic regression analysis exploring the effect of the chosen three “F”-criteria on the presence of definite SUTI or probable SUTI
(Predicitve probability of the logistic regression model: AUC = 0.821 [95% CI 0.755 to 0.887];
Naglerkerke R Squared = 0.454)

|  | p-Value | Odds Ratio | 95% Confidence Interval |
| --- | --- | --- | --- |
| F1 (Fever) | 0.06 | 2.61 | 0.96-7.09 |
| F2 (Failure) | 0.12 | 1.92 | 0.85-4.36 |
| F3 (Focal Symptoms) | <0.01 | 20.34 | 8.56-48.33 |
