# Supplementary material for: A diagnostic algorithm for detection of urinary tract infections in hospitalized patients with bacteriuria: The “Triple F” approach supported by Procalcitonin and paired blood and urine cultures
Source: PLoS One. 2020 Oct 22;15(10):e0240981. doi: 10.1371/journal.pone.0240981 (PMC7580978; doi:10.1371/journal.pone.0240981)
Supplement: S2 Table — (DOCX) [file pone.0240981.s003.docx]

**S2 Table**. Analysis of the association between gender and presence of “F”-criteria and definite diagnoses. P-Values were calculated using the Chi-square test.

| Presence of “F”-criteria or diagnosis | Male (n=92) | Female (n=91) | p-Value |
| --- | --- | --- | --- |
| ABU | 31 | 24 | 0.28 |
| Possible SUTI | 26 | 26 | 0.96 |
| Probable SUTI | 12 | 14 | 0.65 |
| Definite SUTI | 19 | 25 | 0.28 |
| F1 (Fever) | 72 | 68 | 0.57 |
| F2 (Failure) | 57 | 58 | 0.80 |
| F3 (Focal Symptoms) | 27 | 27 | 0.96 |
