# Supplementary material for: A diagnostic algorithm for detection of urinary tract infections in hospitalized patients with bacteriuria: The “Triple F” approach supported by Procalcitonin and paired blood and urine cultures
Source: PLoS One. 2020 Oct 22;15(10):e0240981. doi: 10.1371/journal.pone.0240981 (PMC7580978; doi:10.1371/journal.pone.0240981)
Supplement: S3 Table — (DOCX) [file pone.0240981.s004.docx]

**S3 Table**: Bacteraemia and accordance of paired blood and urine cultures in patients with bacteriuria.
* defined as percentage of accordant bacteraemia of any true bacteraemia for each microorganism

| Microorganism isolated from urine culture | n of cases | Any true bacteraemia | Accordant bacteraemia | Accordance* (%) |
| --- | --- | --- | --- | --- |
| *E. coli* | 94 | 34 | 29 | 85.3% |
| *K. pneumoniae* | 28 | 10 | 5 | 50.0% |
| *P. mirabilis* | 12 | 5 | 3 | 60.0% |
| *P. aeruginosa* | 9 | 2 | 1 | 50.0% |
| *E. faecalis* | 23 | 6 | 1 | 16.7% |
| *E. faecium* | 9 | 2 | 0 | 0.0% |
