# Supplementary material for: A diagnostic algorithm for detection of urinary tract infections in hospitalized patients with bacteriuria: The “Triple F” approach supported by Procalcitonin and paired blood and urine cultures
Source: PLoS One. 2020 Oct 22;15(10):e0240981. doi: 10.1371/journal.pone.0240981 (PMC7580978; doi:10.1371/journal.pone.0240981)
Supplement: S4 Table — F1: fever, F2: failure, F3: focal symptoms. (DOCX) [file pone.0240981.s005.docx]

**S4 Table**. Diagnosis of bacteremic UTI (as surrogate of definite systemic UTI) in relation to “3F” score and PCT. F1: fever, F2: failure, F3: focal symptoms.
Abbreviations: n number; SUTI urinary tract infection with systemic involvement, PCT Procalcitonin, ID infectious disease.

| *“3F“ score* | | | *n (patients)* | *PCT [pg/m]l* | *n (patients)* | *Alternative ID focus* | *Bacteremic SUTI n(%)* |
| --- | --- | --- | --- | --- | --- | --- | --- |
| *F1* | *F2* | *F3* |  |  |  |  |  |
| 1 | 1 | 1 | 30 | ≥ 0.25 | 21 | 3 | 13 (61.9) |
|  |  |  |  | < 0.25 | 9 | 0 | 0 |
| 0 | 1 | 1 | 4 | ≥ 0.25 | 4 | 2 | 0 (0) |
|  |  |  |  | < 0.25 | 0 | 0 | 0 |
| 1 | 0 | 1 | 18 | ≥ 0.25 | 12 | 1 | 6 (50.0) |
|  |  |  |  | < 0.25 | 6 | 2 | 0 |
| 0 | 0 | 1 | 2 | ≥ 0.25 | 1 | 0 | 1 (100.0) |
|  |  |  |  | < 0.25 | 1 | 0 | 0 |
| 1 | 1 | 0 | 58 | ≥ 0.25 | 44 | 19 | 12 (27.3) |
|  |  |  |  | < 0.25 | 14 | 8 | 1 |
| 0 | 1 | 0 | 23 | ≥ 0.25 | 18 | 6 | 5 (27.8) |
|  |  |  |  | < 0.25 | 5 | 2 | 0 |
| 1 | 0 | 0 | 34 | ≥ 0.25 | 22 | 10 | 5 (22.7) |
|  |  |  |  | < 0.25 | 12 | 5 | 1 |
| 0 | 0 | 0 | 14 | ≥ 0.25 | 8 | 6 | 0 (0) |
|  |  |  |  | < 0.25 | 6 | 3 | 0 |
