# Supplementary material for: A diagnostic algorithm for detection of urinary tract infections in hospitalized patients with bacteriuria: The “Triple F” approach supported by Procalcitonin and paired blood and urine cultures
Source: PLoS One. 2020 Oct 22;15(10):e0240981. doi: 10.1371/journal.pone.0240981 (PMC7580978; doi:10.1371/journal.pone.0240981)
Supplement: S5 Table — (DOCX) [file pone.0240981.s006.docx]

**S5 Table**. Application of the “3F” algorithm as an antimicrobial stewardship (ABS) tool.
Abbreviations: ABU asymptomatic bacteriuria, UTI urinary tract infection, SUTI urinary tract infection with systemic involvement, ABS antimicrobial stewardship, ID infectious disease.

|  | Withholding antimicrobial therapy possible | | Antimicrobial therapy with narrower spectrum possible | |
| --- | --- | --- | --- | --- |
|  | Threshold to treat: possible UTI | Threshold to treat: probable UTI | Threshold to treat: possible UTI | Threshold to treat: probable UTI |
| ABU *without* alternative ID  (n of patients) | 14 | 14 | - | - |
| ABU + alternative ID (n of patients) | - | - | 41 | 41 |
| Possible SUTI *without* alternative ID  (n of patients) | 0 | 31 | - | - |
| Possible SUTI + alternative ID focus  (n of patients) | - | - | 0 | 21 |
| Eligible for ABS intervention  n(% of patients with bacteriuria) | 14 (7.7) | 45 (24.6) | 41 (22.4) | 62 (33.9) |
